# Comparative efficacy of nerve coagulation vs. cap implantation in neuroma prevention using the rat sciatic nerve model: A histological and neuroradiological insight

**DOI:** 10.1371/journal.pone.0355057

**Published:** 2026-08-14

**Authors:** Martin Aman, Tess T. Klemm, Maximilian Mayrhofer-Schmid, Adriana C. Panayi, Merle Brunnée, Daniel Schwarz, Leila Harhaus, Arne H. Boecker

**Affiliations:** 1 Department of Hand-, Plastic and Reconstructive Surgery, Burn Center, BG Trauma Center Ludwigshafen, Department of Hand- and Plastic Surgery, University of Heidelberg, Heidelberg, Germany; 2 Department of Hand, Replantation, and Microsurgery BG Klinikum Unfallkrankenhaus Berlin and Chair of Hand, Replantation, and Microsurgery at the Charité University Medicine Berlin; 3 Charité – Universitätsmedizin Berlin, corporate member of Freie Universität Berlin, Humboldt-Universität zu Berlin, and Berlin Institute of Health, Department of Oral and Maxillofacial Surgery, Berlin, Germany; 4 Department of Neuroradiology, Heidelberg University Hospital, Heidelberg, Germany; Suez Canal University Faculty of Medicine, EGYPT

## Abstract

**Introduction:**

Peripheral nerve injuries pose a considerable challenge in reconstructive surgery due to the risk of neuroma formation, often resulting in severe pain and significant functional impairment. Various treatment modalities, including nerve coagulation and the use of nerve caps, have been explored to manage neuroma development. In this study, we compare the efficacy of chitosan and polylactide nerve caps to standard nerve coagulation in modulating neuroma morphology and promoting organized nerve regeneration.

**Methods:**

Female Sprague Dawley rats (n = 40) were divided into four equal groups: Sham (control), Coagulation, Polylactide Cap (Neurocap®), and Chitosan Cap. After neurotmesis of the sciatic nerve, the respective treatments were applied. The evaluation period spanned 12 weeks, with MR neurography performed at 6 and 12 weeks. Histological analyses of the harvested tissue were performed at 12 weeks. A survey among 16 plastic and nerve surgeons assessing neuroma formation and scarring was conducted.

**Results:**

On macroscopic evaluation, the Chitosan (C) and Polylactide (P) Cap groups displayed denser, more contained neuromas, while the Sham (S) and Coagulation (K) groups exhibited irregularly shaped neuromas with peripheral extensions. Histomorphometry showed higher axon density [ρ(C) = 15851 ± 7132 axons/mm², ρ(P) = 15657 ± 4066 axons/mm²] and thicker myelin sheaths [thm(C) = 0.808 ± 0.054 µm, thm(P) = 0.786 ± 0.045 µm] in the cap-treated groups compared to Sham [ρ(S) = 8052 ± 5080 axons/mm², thm(S) = 0.704 ± 0.060 µm; p(C/S) = 0.0193, p(P/S) = 0.0029 for axon density, p(C/S) = 0.0019, p(P/S) = 0.0046 for myelin thickness], but with no significant differences compared to the Coagulation group [ρ(K) = 14023 ± 7383 axons/mm², thm(K) = 0.745 ± 0.075 µm, p > 0.05]. Surgeon survey findings aligned with macroscopic evaluations, indicating consistency in neuroma shape classification.

**Conclusion:**

Chitosan and polylactide nerve caps effectively promote organized nerve regeneration and prevent disorganized neuroma formation. Nerve coagulation demonstrated comparable efficacy in certain parameters, suggesting it could remain a viable treatment option. These findings provide valuable insights for optimizing surgical approaches to peripheral nerve injuries, paving the way for more personalized and effective therapeutic strategies.

## Introduction

Peripheral nerve injury, which accounts for approximately 3% of all trauma cases, presents a significant challenge in the field of reconstructive surgery [1]. Complete severance of a nerve, if not promptly and adequately addressed, can lead to unguided axonal regeneration, resulting in neuroma formation and culminating in functional impairments and severe pain [2]. Post-traumatic neuroma development has a reported average incidence rate of 13–19%, with approximately 7% occurring specifically after finger amputations, underscoring the clinical need for effective management strategies [3–5].

Neuromas represent a failed attempt at regeneration in response to nerve injury, characterized by the disorganized proliferation of nerve fibers attempting to regenerate in the absence of a direct pathway to their target tissues. This aberrant regrowth results in a tangled, disorganized mass of nerve fibers, collagen, and surrounding tissue, and can lead to significant pain, sensitivity, and functional impairment. The process is driven by a complex interplay of factors, including inflammation, Schwann cell proliferation, and the extracellular matrix environment, which collectively disrupt normal nerve regeneration mechanisms [2,6,7]. Clinically, neuromas can significantly impact quality of life, manifesting as spontaneous pain, hyperalgesia, and allodynia [8]. Moreover, mechanical pressure and traction on the neuroma can exacerbate symptoms, complicating the management of this condition [7,9].

To date, a diverse array of treatment modalities has been explored, ranging from conservative measures such as stump desensitization and adapted pharmacological therapy to surgical interventions, including nerve coagulation, shortening, and encapsulation within surrounding soft tissue, veins, or dermal layers. However, the efficacy and superiority of these approaches remain subjects of ongoing debate within the literature [10–14].

Despite the various treatments available for neuroma management, their efficacy has been inconsistent. Surgical removal of neuromas, while offering temporary relief, does not always prevent recurrence and may even lead to the formation of a larger, more painful neuroma at the surgical site [15,16]. Non-surgical treatments, such as steroid injections and nerve blocks, provide transient relief but fail to address the underlying pathophysiology of neuroma formation [17]. The challenge lies in creating an environment that supports organized nerve regeneration while minimizing the potential for painful neuroma development.

Recent advancements in biomaterials have led to the development of artificial nerve caps designed to encapsulate the nerve end, guide nerve regeneration, and prevent the formation of neuromas [18]. These devices, crafted from biocompatible materials such as chitosan and polylactide, act as conduits for nerve growth while providing a physical barrier against the disorganized proliferation of nerve fibers [19,20]. Chitosan-based nerve caps, in particular, offer a unique advantage due to their inherent properties that support nerve regeneration. Chitosan has been shown to promote Schwann cell proliferation and alignment, which are essential for guiding regenerating axons towards their target [21–23]. Additionally, the biodegradable nature of chitosan suggests that the cap gradually dissolves as the nerve heals, reducing the need for a second surgery to remove the device [24,25].

This study aims to investigate the efficacy of a novel chitosan-based nerve cap in the prevention of unorganized neuroma formation following traumatic nerve lesions, comparing its performance against both the standard surgical treatment—nerve shortening and coagulation—and a polylactide-based nerve cap. The latter, commercially available as Neurocap® from Polyganics, acts as a mechanical barrier to regenerating axons without influencing neuroma formation at a biochemical level [26]. Through this comparative analysis, we seek to elucidate the potential of nerve caps in general for preventing unorganized neuroma formation and the effect of chitosan on axonal sprouting in particular, thereby contributing valuable insights for the optimization of surgical treatments for neuroma prevention.

## Methods

### Animal model and group assignment

Forty female Sprague Dawley rats (mean weight 220g ± 30g) were housed and treated in strict accordance with FELASA principles and under the animal experiment permit issued by the State Investigation Office Koblenz (reference number 23 177–07/G 21-7-045). The animals were randomized into four groups (n = 10 per group):

1. Sham: Neurotmesis without any treatment, followed by skin closure.
2. Coagulation: Neurotmesis followed by nerve shortening and coagulation.
3. Polylactide Cap: Neurotmesis followed by nerve shortening and application of a nerve cap composed of the copolymer poly(DL-lactide-ԑ-caprolactone) (Neurocap®).
4. Chitosan Cap: Neurotmesis followed by nerve shortening and application of a chitosan-based nerve cap.

One Chitosan Cap animal died under anesthesia during the first MRI (6 weeks post-operatively) and was excluded from all subsequent analyses. For histomorphometry, one specimen per group was allocated to qualitative longitudinal histology, which required separate tissue preparation and could not also undergo osmium-based transverse histomorphometry, reducing each group by one. In the MRI cohort, four Sham animals were not imaged owing to limited scanner availability, with scanning prioritized for the intervention groups, and one Polylactide Cap MRI dataset was excluded due to file corruption. The resulting numbers per analysis are given in Table 1.

**Table 1:** Animals per analysis.

Analysis	Sham	Coagulation	Chitosan Cap	Polylactide Cap
MRI (6 & 12 weeks)	6	10	9	9
Histomorphometry	9	9	8	9
Systematic macroscopic evaluation	10	10	9	10

Number of animals included per statistical analysis. Qualitative longitudinal histology sections are not listed, as they were assessed descriptively with one representative specimen per group and did not enter statistical analysis. x

### Surgical procedure and evaluation timeline

After a 10-day acclimatization period, surgical interventions were performed under isoflurane-oxygen inhalation anesthesia (Isofluran CP 1 ml/mg, CP-Pharma Handelsgesellschaft mbH, Burgdorf, Germany). Anesthesia was induced with 5% isoflurane in oxygen at a flow rate of 500 ml/min and maintained with 1.5–2.5% isoflurane at the same flow rate. The depth of anesthesia and surgical tolerance were assessed by testing the interdigital reflex and adjusted perioperatively according to the respiratory rate.

For peri- and postoperative pain management, buprenorphine (0.04 mg/kgBW; Buprenovet Multidose 0.3 mg/ml, VetViva Richter GmbH, Wels, Austria) was administered at the start of every surgery. A skin incision along the thigh exposed the sciatic nerve, followed by neurotmesis and excision of a 5 mm nerve segment. One of the four treatment interventions was then applied. The caps were secured using microsurgical simple interrupted sutures (Ethilon^TM^ (Polyamide) 9−0, Johnson & Johnson Medical GmbH, Norderstedt, Germany).

Postoperatively, rats were closely monitored for signs of pain or distress. Analgesics (buprenorphine 0.04 mg/kgBW) were administered every 8 hours for seven days, with additional doses given if required. All efforts were made to minimize suffering.

The evaluation period spanned 12 weeks. MR neurography was conducted at 6 and 12 weeks post-surgery to assess neuroma development and cap degeneration. At 12 weeks, animals were euthanized under anesthesia (induction: 5% isoflurane, 500 ml/min O₂; maintenance: 1.5–2.5% isoflurane, 500 ml/min O₂) with an overdose of pentobarbital sodium (3.5 ml/kgBW Narcoren® 16 g/100 ml, Boerhringer Ingelheim Vetmedica GmbH, Ingelheim, Germany). The treated nerve segments were then photographed and harvested for histological analysis.

### MRI assessment

Each animal in the intervention groups and six randomized animals from the Sham group underwent MRI imaging at two time points (6 and 12 weeks after surgery). For the MRI, the animals were anesthetized with 3% isoflurane in oxygen at a flow rate of 500 ml/min for induction and maintained with 1–2% isoflurane throughout the procedure. The animals were positioned supine on a heating pad, and respiratory rate was continuously monitored using a breathing surface pad connected to a custom-written LabView program (National Instrument Corporation, Austin, TX, USA) [27].

MRI imaging was performed using a 9.4 T horizontal bore small animal scanner (BioSpec 94/20, Bruker Biospin MRI, Ettlingen, Germany) equipped with a four-channel phased-array surface receiver coil. The MRI protocol included a high-resolution T2-w Rapid Acquisition with Refocused Echoes (RARE) sequence with flip-back technique: 2D sequence, echo time (TE): 40 ms, repetition time (TR): 2500 ms, rare factor 4, spectral fat saturation, acquisition matrix: 345 × 150, number of slices: 23, slice thickness: 1 mm, number of averages: 1, flip angle: 90°.

### Histological processing

Myelin and axonal structures were stained to quantify nerve regeneration and neuroma formation. Nerve specimens (proximal ends, ~ 1 cm in length) were fixed in formalin for 24 hours and rinsed in phosphate buffer solution. From each group, one specimen was reserved for qualitative longitudinal histology and directly dehydrated and embedded in paraffin, while the rest werefirst treated with osmium tetroxide staining for myelin visualization and subsequent quantitative transverse histomorphometric analysis before dehydrating and paraffin embedding. Transverse sections (2 µm thick) were prepared to analyze axon density, axon diameter, myelin sheath thickness and distribution, while longitudinal sections (5 µm thick) were used to evaluate neuroma formation. Masson’s Trichrome and Hematoxylin and Eosin (H.E.) staining were performed on the longitudinal sections to examine regenerating axon conformity and scar tissue development.

Quantitative analysis was conducted to assess axon number, density, and size, as well as myelin sheath thickness. Using ImageJ Version 2.3.0/1.53q (Rasband, W.S., ImageJ, National Institutes of Health, Bethesda, Maryland, USA, https://imagej.nih.gov/ij/) the diameter of each specimen was measured. Sampling fields were selected using the “systematic random sampling” principle outlined by Guena et al. [28,29]. Axons were counted and measured both with and without their myelin sheath, and myelin sheath thickness was subsequently calculated. Axon density (n/mm²) was calculated by dividing the number of axons counted in the sampling fields by the total area of these fields. The relationship between axon and total fiber thickness was calculated as g-ratio and the total axon count was estimated by multiplying the average axon density by the total cross-sectional area of the nerve.

### Quantitative analysis of cap degradation and neuroma shape

Cap degradation was analyzed semi-quantitatively. Two radiologists independently evaluated T2-weighted MRI images of the nerve ends and classified cap degradation for the chitosan- and polylactide caps on a Likert scale (0: complete degradation; 1: rather degradated; 2: rather not degradated; 3: no degradation). Interrater reliability was calculated using Cohen’s kappa coefficient.

The maximal diameter of the neuroma was determined using longitudinal T2-weighted MRI images. Neuroma length was measured from the most distal point of the maximal diameter to the neuroma’s endpoint. Neuroma boundaries were defined as regions exceeding 1.4 times the T2-signal intensity of the adjacent normal nerve tissue. Neuroma shape was quantified using Fiji ImageJ software by calculating circularity according to the formula $\text{4}\pi \times (area/perimeter^{\text{2}})$. A value of 1.0 indicates a perfect circle, while values approaching 0.0 indicates an increasingly elongated shape.

### Systematic macroscopic evaluation

**Fig 1:**
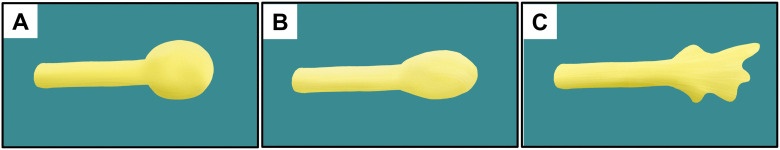
Neuroma shape categories. Approximation of the three neuroma shape categories proposed by Raasveld et al., which were applied in this study for evaluation. The images depict a bulbous neuroma (A), a fusiform neuroma (B), and an atypical neuroma (C).

Macroscopic evaluation assessed neuroma shape, adhesions, and cap degeneration. Sixteen plastic and nerve surgeons, blinded to the experimental group assignments, evaluated neuroma formation and shape based on Raasveld et al.’s classification (Fig 1), as well as perineural scarring following previously outlined criteria: score 0 – absent or thin, 1 – mild, 2 – moderate and 3 – severe [30,31]. Surgeons were presented with images of the nerve ends in random order and asked to classify neuroma shape and severity of scarring into these predefined categories. Interrater reliability was calculated using Fleiss’ kappa [32,33].

### Data analysis

MRI data was processed using high-resolution anatomical and functional sequences to assess nerve and neuroma structure and function. Macroscopic assessment categorized neuroma shape and adhesion levels according to Raasveld et al. and Yamamoto et al., respectively [30,31]. Histological analysis involved fixation, staining, and preparation of transverse and longitudinal nerve sections. Axon density, axon diameter, myelin sheath thickness and g-ratio were quantified in image sections chosen using systematic random sampling.

Statistical analysisStatistical analyses were performed using one-way ANOVA and two-sided t-tests to compare groups [34] with Prism 5 (GraphPad Software, San Diego, CA, USA). Normal distribution was confirmed using Shapiro-Wilk, while homogeneity of variances was evaluated with the F-test. Interrater reliability of the survey responses and MRI analyses were assessed in SPSS (IBM SPSS Statistics for Windows, Version 29.0, IBM Corp, Armonk, NY, USA) using Fleiss’ kappa and Cohen’s kappa, respectively. Post-hoc effect size and power calculations were performed in RStudio (R version 4.3.2) using the pwr package. To evaluate the magnitude of observed effects, Cohen’s f was calculated for ANOVA comparisons, while Cohen’s d was calculated for the pairwise t-tests. Statistical significance was set at p < 0.05.

## Results

### Neuroma formation and adhesion

**Fig 2:**
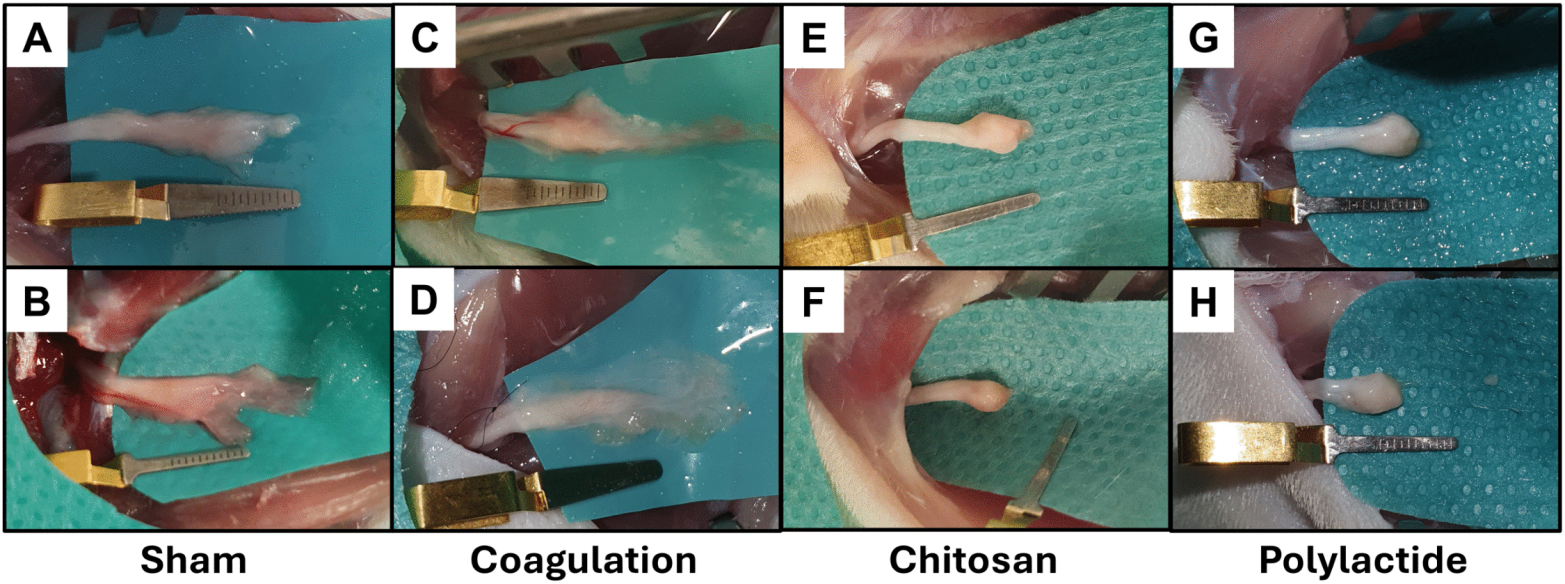
Macroscopic findings. Two representative images of the neuroma in each group during the explantation in week 12, after being freed from surrounding scar tissue in situ.

Macroscopic evaluation showed that the Sham and Coagulation groups had irregularly shaped neuromas with peripheral extensions, making them difficult to separate from adjacent scar tissue. In contrast, the Chitosan and Polylactide Cap groups produced denser neuromas without peripheral extensions (Fig 2). Caps remained identifiable at 12 weeks, surrounded by scar tissue.

**Fig 3:**
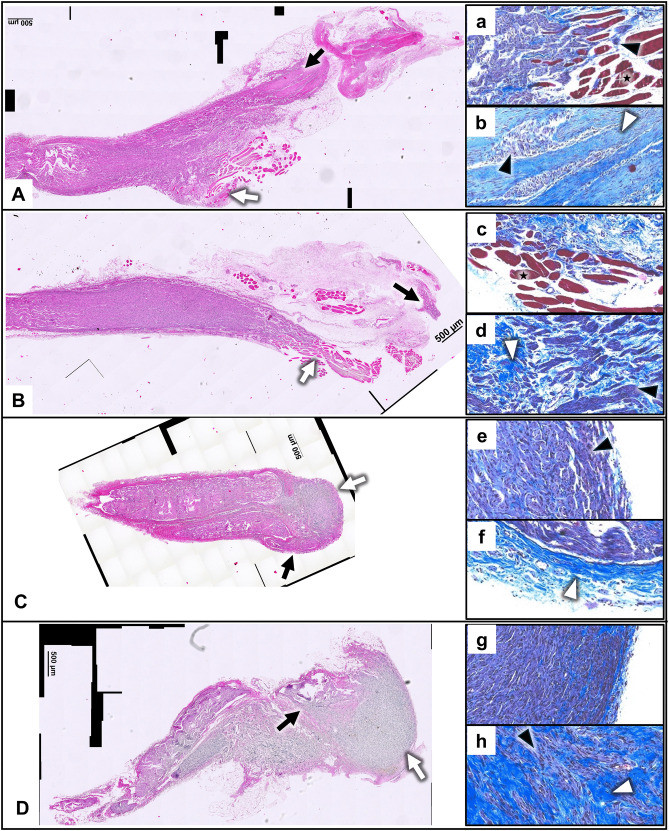
Histological evaluation of longitudinal sections across experimental groups. Sham (A), Coagulation (B), Chitosan Cap (C), Polylactide Cap (D). Scale bars = 500 µm. Left column: Hematoxylin and Eosin (H&E) stained longitudinal sections for each group, providing an overview of overall neuroma architecture and enabling size comparison between groups. In the H&E stain, cell nuclei appear blue-violet, while cytoplasm, collagen fibers, and remaining tissue components appear pink. Arrows indicate the regions selected for higher-magnification Masson’s Trichrome insets: white arrows correspond to the upper insets (a, c, e, g) and black arrows to the lower insets (b, d, f, h). Right column: Corresponding higher-magnification insets stained with Masson’s Trichrome, highlighting the distribution of fibrosis and axonal organization. In the Masson’s Trichrome stain, collagen appears blue (white arrowheads), nerve tissue and perineural cells appear with red-violet cytoplasm and black-brown cell nuclei (black arrowheads), and skeletal muscle appears red (black star). The complete Massons’s Trichrome stained longitudinal sections for each group can be found in the supporting information S1 Fig.

Qualitative histological assessment of the longitudinal sections offered tentative support for some of these findings. In the Sham group, progressive loss of parallel fiber organization was observed (Fig 3A), with nerve fascicles extending into surrounding muscle and adipose tissue (Fig 3a) and increasing intermingling of collagen and nerve fibers distally (Fig 3b). The Coagulation group similarly showed loosening of fiber arrangement, though to a lesser degree (Fig 3B). The Cap groups demonstrated bulbous, relatively well-demarcated neuroma endings with dense nerve tissue and little evidence of infiltration into surrounding structures (Fig 3C, D). In the Chitosan Cap group, connective tissue deposition was predominantly perineural rather than intraneural (Fig 3f). In the Polylactide Cap group, the neuroma ending appeared well-demarcated with minimal intraneural connective tissue (Fig 3g), though a degree of intraneural connective tissue deposition was observed more proximally within the nerve stump (Fig 3h).

### Cap degradation

**Fig 4:**
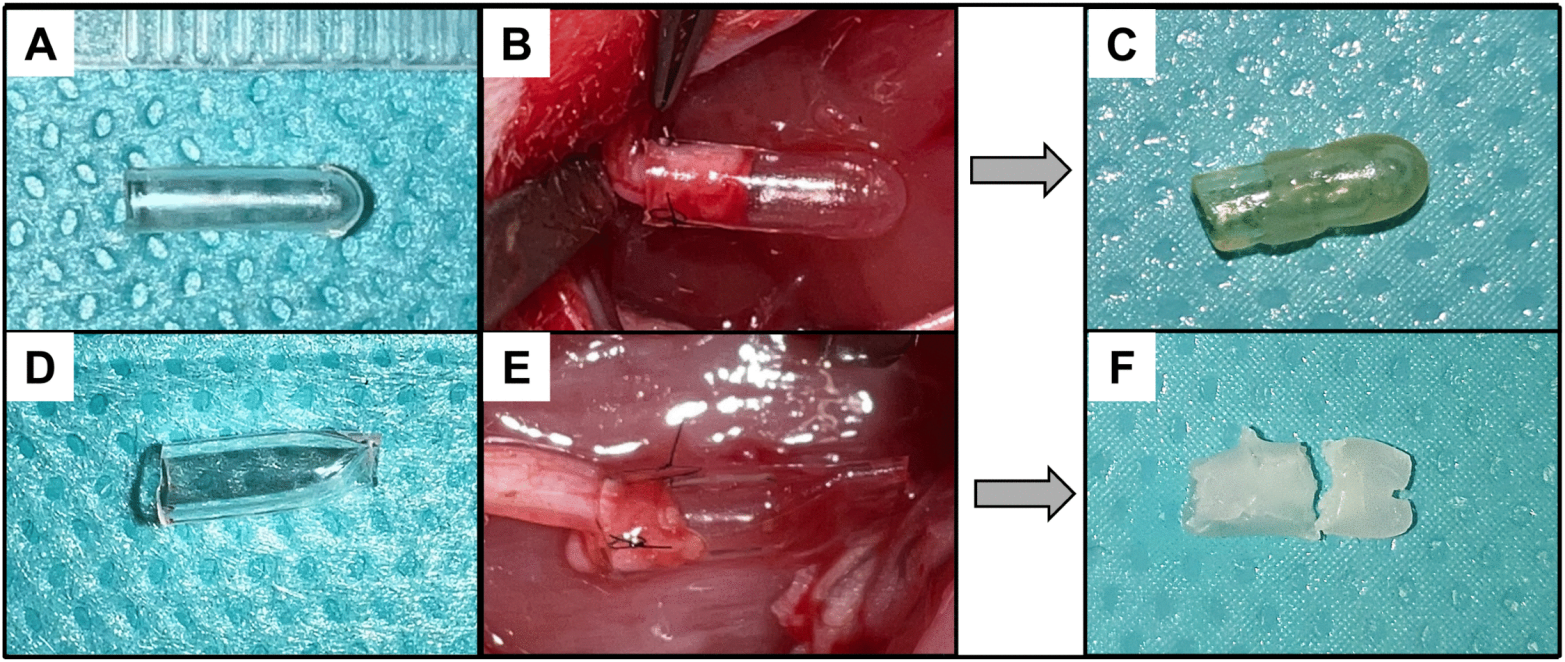
Cap degradation. Shown are representative images in chronological order illustrating the degradation of the chitosan cap (top row) and the polylactide cap (bottom row). In the first column, a trimmed chitosan cap (A) and polylactide cap (D) can be seen shortly before implantation. Caps made of the same material are shown in B and E, implanted after neurotmesis. In the final column, two explanted caps (C, F) are shown after the 12-week observation period.

Chitosan caps showed minimal degradation, remaining hard and stable, with occasional tearing at the suture site and discoloration. Polylactide caps exhibited more pronounced degradation, becoming indented, brittle, and changing from transparent to opaque white (Fig 4).

**Fig 5:**
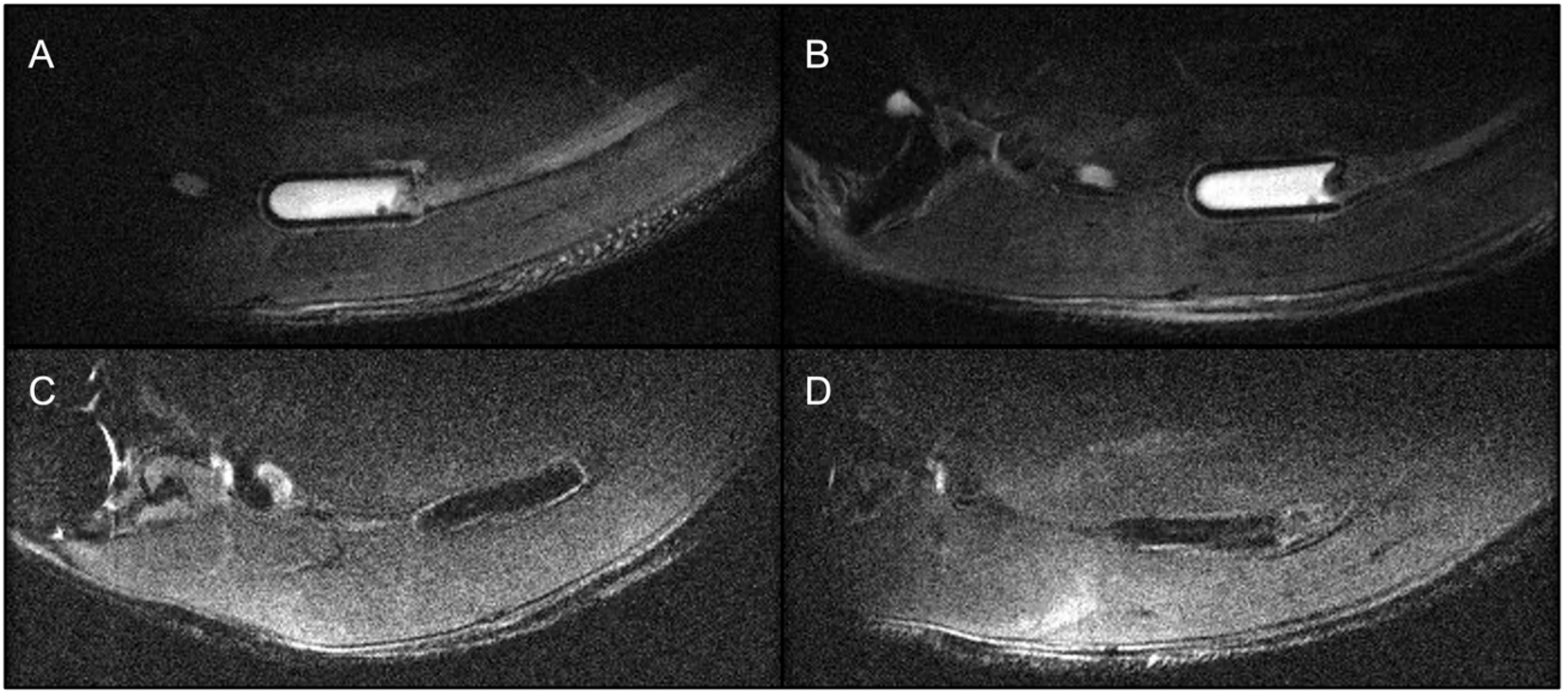
MRI evaluation of cap degradation. T2-weighted MRI images showed that chitosan caps displayed hardly any degradation from 6 weeks (A) and 12 weeks (B) after surgery. The polylactide caps showed little degradation from 6 weeks (C) to 12 weeks (D) after surgery.

The semiquantitative analysis of cap degradation based on the MRI scans showed that chitosan caps exhibited minimal degradation, with no significant changes between the first MRI after 6 weeks and the second MRI after 12 weeks (3.0 ± 0, p > 0.99). Interrater agreement was perfect, with a Cohen’s kappa = 1. The polylactide caps showed little degradation, visible in the MRI as minor cap deformity (2.61 ± 0.5, p > 0.99). Interrater agreement was fair, with a Cohen’s kappa = 0.2 (Fig 5).

### Histomorphometry

Significant differences in axon density (ρ) and myelin sheath thickness (thm) were observed. The Chitosan and Polylactide groups had denser neuromas [ρ(C) = 15851 ± 7132 axons/mm², ρ(P) = 15657 ± 4066 axons/mm²] and thicker myelin sheaths [thm(C) = 0.808 ± 0.054 µm, thm(P) = 0.786 ± 0.045 µm] compared to the Sham group [ρ(S) = 8052 ± 5080 axons/mm², thm(S) = 0.704 ± 0.060 µm; p(C/S) = 0.0193, p(P/S) = 0.0029 for axon density, p(C/S) = 0.0019, p(P/S) = 0.0046 for myelin thickness]. Effect size analysis indicated large differences for these comparisons (Cohen’s d: axon density ρ(S/C) = 1.27 and ρ(S/P) = 1.65, myelin sheath thickness thm(S/C) = 1.82) and thm(S/P) = 1.55, with observed post-hoc power at 66–92%, supporting the robustness of these findings.

**Fig 6:**
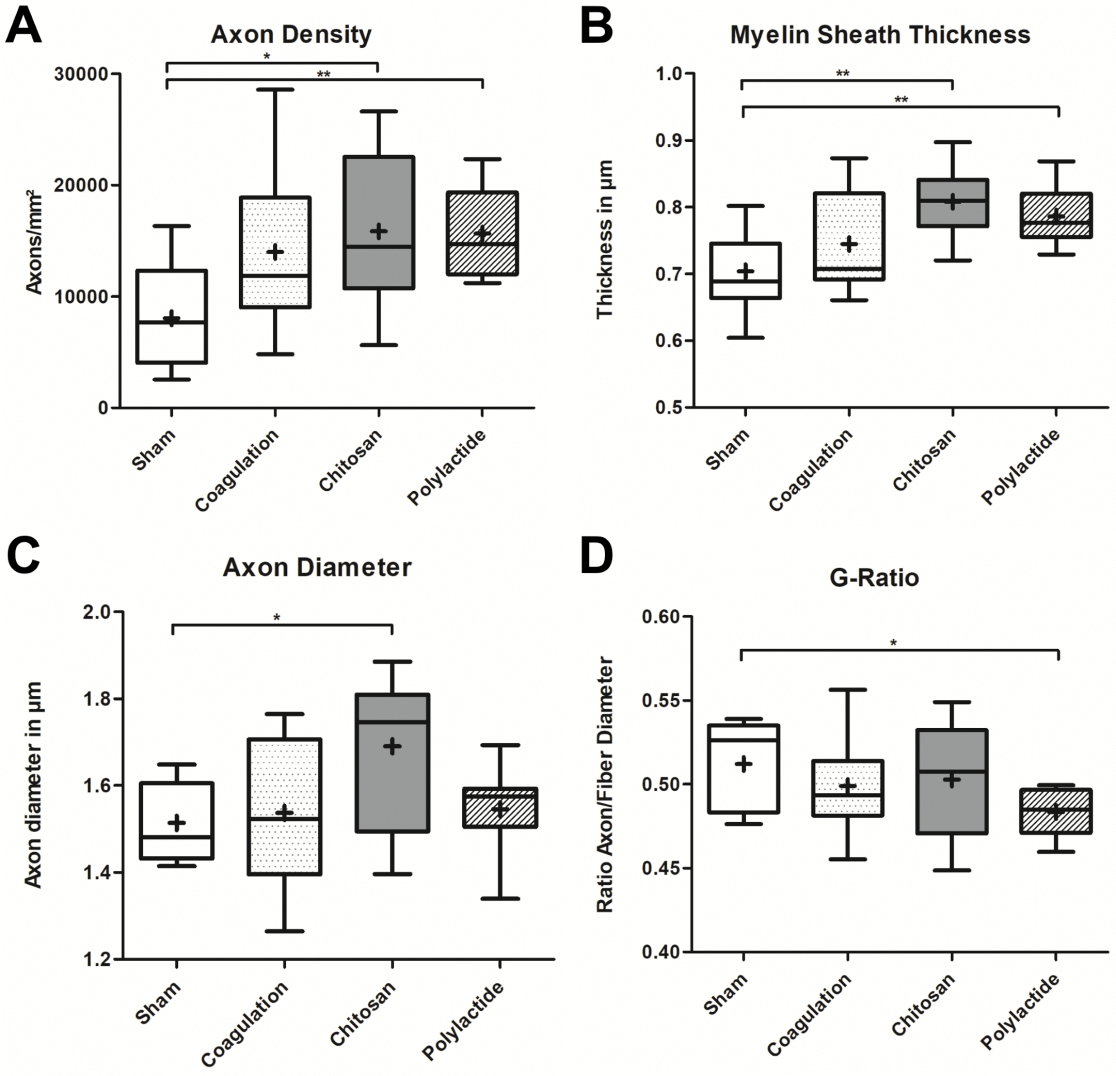
Histomorphometric results. All histomorphometric results are shown in a boxplot, with the arithmetic mean marked as a “+” and the whiskers representing the full range. Significances are indicated as *p < 0.05 and **p < 0.01.

**Fig 7:**
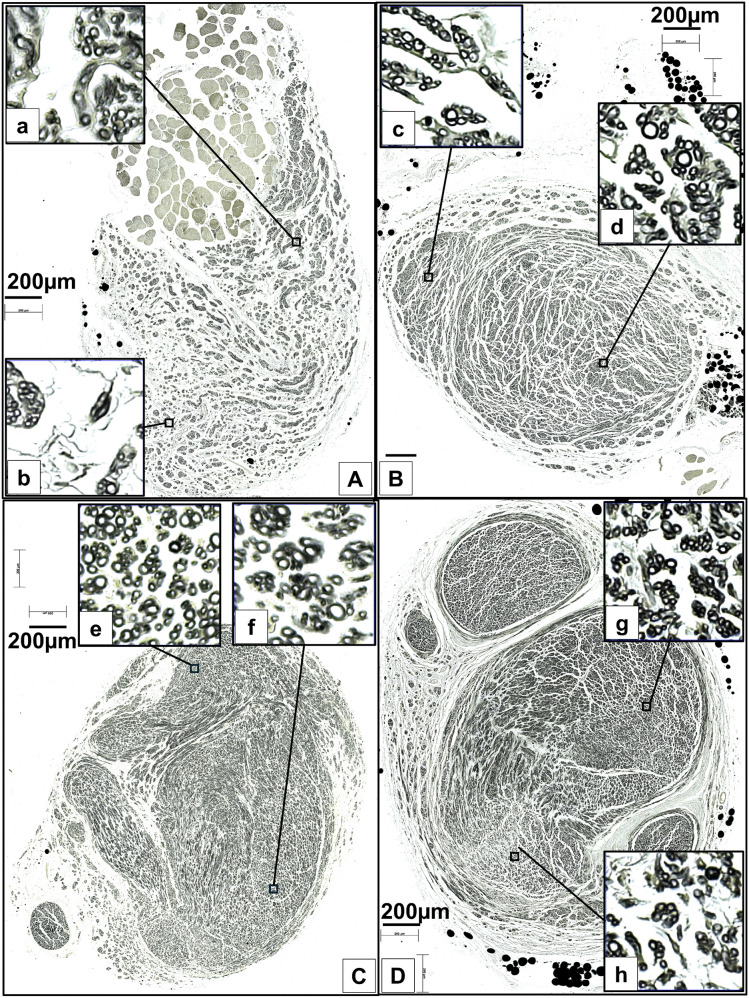
Histomorphometric evaluation of transverse sections across experimental groups. Sham (A), Coagulation (B), Chitosan Cap (C), Polylactide Cap (D). Scale bars = 200 µm were added for readability during figure preparation based on the original calibrated scale bars embedded by the imaging software, which remain visible. High-magnification examples (a–h; scale: 50 × 50 µm) of transverse sections used in the histomorphometric evaluation, illustrating overall axonal density, axon diameter, and myelin sheath thickness. Myelinated axons appear as dark-staining, ring-shaped profiles, with the osmium tetroxide–stained myelin sheath (black) surrounding the unstained axoplasm (white). The complete set of transverse sections for each group is provided in the supporting information (S2 Fig–S5 Fig).

No significant differences were found between the Cap groups and the Coagulation group [ρ(K) = 14023 ± 7383 axons/mm², thm(K) = 0.745 ± 0.075 µm, p > 0.05], where effect sizes were smaller (Cohen’s d < 0.96) and power medium to low (8–43%). Differences in axon diameter (da) were significant between the Chitosan Cap [da(C) = 1.690 ± 0.182] and Sham [da(S) = 1.514 ± 0.090] groups (p = 0.021, Cohen’s d = 1.25, power = 65%), and in g-ratio (g) between the Polylactide Cap [g(P) = 0.484 ± 0.014] and Sham (g(S) = 0.521 ± 0.026) groups (p = 0.0112, Cohen’s d = 1.35, power = 77%), with no significant differences, as well as low power and effect sizes (Cohen’s d < 1 and power < 47%), among the other groups (Figs 6 and 7).

### Survey results

A survey among 16 plastic and nerve surgeons assessed neuroma formation, shape, and scarring around the nerve according to Raasveld et al. and Yamamoto et al. categories [30,31].

The results of the survey showed that the surgeons classified neuroma shapes and adhesion grades similarly to the macroscopic findings.

Interrater reliability for neuroma shape classification indicated a moderate level of agreement (κ = 0.587, p < 0.001). The majority of neuromas in the Polylactide (73%) and Chitosan Cap (66%) groups were classified as bulbous, whereas neuromas in the Sham and Coagulation groups were predominantly categorized as atypical in 71% and 86% of cases, respectively.

### Neuroma analysis (MRI)

Analysis of neuroma shapes on T2-weighted MRI images revealed distinct patterns among the groups. Neuromas in the Sham and Coagulation groups exhibited an elongated triangular shape with low sphericity values (Sham: 0.283 ± 0.066 vs. Coagulation: 0.382 ± 0.077, p = 0.0006). In contrast, neuromas in the Chitosan and Polylactide Cap groups were shaped like a half-circle, showing moderate sphericity values (Chitosan: 0.706 ± 0.066 vs. Polylactide: 0.753 ± 0.047, p = 0.015).

**Fig 8:**
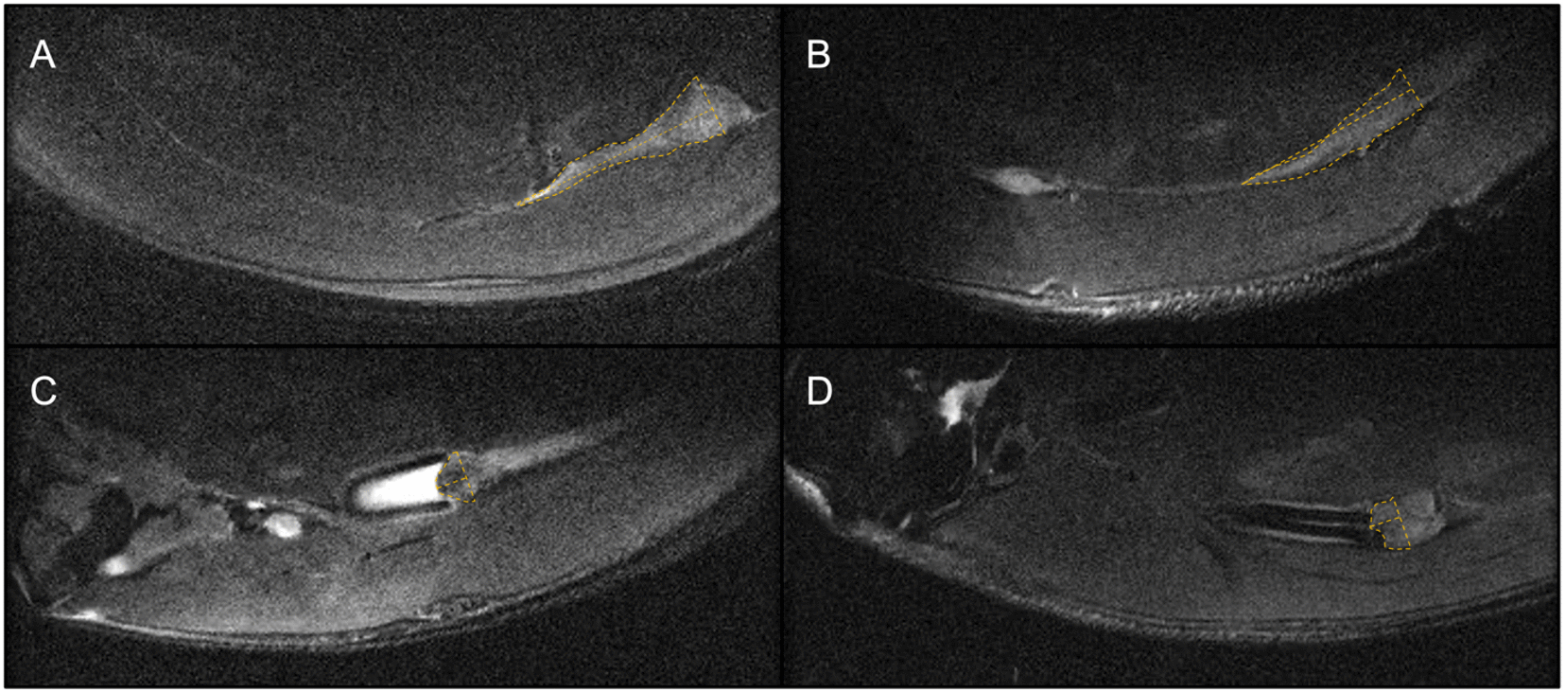
MRI visualization of neuroma formation. Neuromas in the Sham (A) and Coagulation (B) groups exhibited an elongated triangular shape, whereas the neuromas in the Chitosan (C) and Polylactide (D) groups displayed a semi-circular shape. The yellow dashed lines represent the regions of interest (ROIs) used to measure the neuroma sphericity, maximum diameter and length.

**Fig 9:**
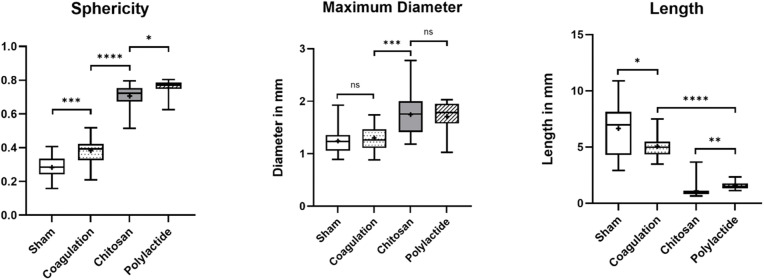
Neuroma sphericity, diameter, and length. Measurements were based on longitudinal T2-weighted MRI images. *, p < 0.05; **, p < 0.005; ***, p < 0.0005; ****, p < 0.00005; ns, not significant.

The maximum diameters of the neuromas were lower in the Sham (1.245 ± 0.271 mm) and Coagulation groups (1.304 ± 0.231 mm) compared to the Chitosan (1.746 ± 0.401 mm, p = 0.0002) and Polylactide Cap groups (1.712 ± 0.301 mm). However, due to their elongated triangular configuration, neuroma lengths were significantly greater in the Sham (6.646 ± 2.363 mm) and Coagulation groups (5.060 ± 0.998 mm) compared to the Chitosan (1.078 ± 0.632 mm) and Polylactide Cap groups (1.573 ± 0.330 mm, p < 0.0001) (Figs 8 and 9).

### Adhesion

**Fig 10:**
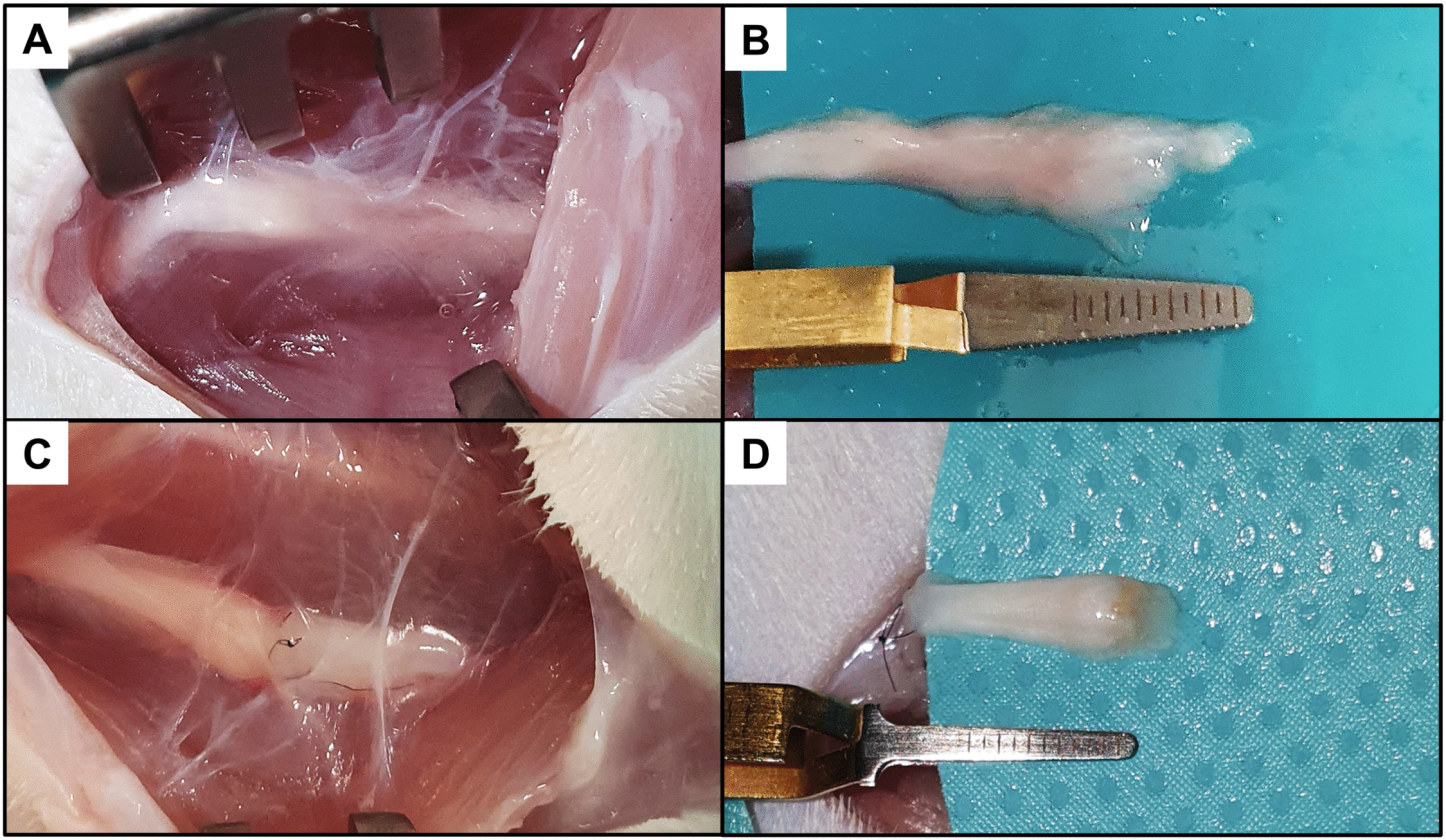
Macroscopic evaluation. Representative images of the nerves in vivo. Images were taken during the explanation of SD21K and SD31P to assess perineural adhesion (A + C) and neuroma shape (B + D). 5 mm ruler with 0.5 mm markings is included for scale.

The Fleiss’ kappa value for differentiation of perineural adhesions from images was κ = 0.137 (p < 0.001). Moderate adhesions (grade 2) were reported in 39–49% of cases in the Sham, Coagulation, and Polylactide Cap groups, while severe adhesions (grade 3) were observed in 42% of cases in the Chitosan Cap group (Table 2). Nerve endings were more easily separable from the surrounding tissue in the Chitosan and Polylactide Cap groups. The Sham and Coagulation groups displayed direct connective tissue attachment to the nerves, with axonal outgrowths into surrounding connective and muscle tissue (Fig 10).

**Table 2:** Adhesion survey results.

Groups	Sham	Coagulation	Chitosan Cap	Polylactide Cap
**Number of Responses**	160	144*	144	160
**Answer Distribution**				
No/Barely (0)	16 (10)	7 (5)	9 (6)	13 (8)
Light (1)	56 (35)	39 (27)	18 (13)	41 (26)
Moderate (2)	62 (39)	70 (49)	56 (39)	64 (40)
Severe (3)	26 (16)	28 (19)	61 (42)	42 (26)
**Mode**	Moderate (2)	Moderate (2)	Severe (3)	Moderate (2)

Results presented as n (%), unless otherwise stated.

* Results of the Sham and Polylactide Cap groups are based on images from 10 animals. Results of the Coagulation and Chitosan Cap groups are based on images from 9 animals, as in the Chitosan Cap group, one animal was excluded due to premature death in week 6, and in the Coagulation group, one set of images was excluded due to quality issues.

## Discussion

This study provides novel insights into the efficacy of chitosan and polylactide nerve caps compared to the standard nerve coagulation method in modulating neuroma formation and promoting organized nerve regeneration following traumatic nerve injury. Our findings reveal that both chitosan and polylactide caps offer distinct advantages, though each comes with unique considerations that may influence clinical decision-making. Additionally, our data suggests that nerve coagulation may be as effective as nerve caps in certain aspects, presenting an intriguing area for further discussion and comparison.

### Neuroma formation

Macroscopic evaluation showed that both chitosan and polylactide nerve caps result in denser neuromas without peripheral extensions, as opposed to the irregularly shaped neuromas with axonal outgrowth seen in the Sham and Coagulation groups. The exact working mechanism of nerve caps has not yet been definitively elucidated, but this finding agrees with suggestions in current literature that say organized nerve regeneration can be facilitated by physical barriers that guide axonal growth and decrease adhesion to surrounding tissue [19,20]. The lack of peripheral sprouting in the cap-treated groups suggests a more restricted and potentially less painful neuroma, which could translate to better clinical outcomes for patients [19,31].

Interestingly, while the neuroma morphology differed between the Coagulation and Cap groups, the histomorphometric results suggest that the coagulation method is not necessarily inferior. This suggests that nerve coagulation, despite its simpler approach, may be comparably effective in managing neuroma formation. Further studies with larger sample sizes are needed to draw definitive conclusions.

### Cap degradation

The observed degradation patterns of the caps show important trends for understanding their long-term functionality and biocompatibility. Chitosan caps showed minimal degradation, maintaining structural integrity over 12 weeks. This prolonged stability could ensure continued protection against neuroma formation but raises concerns about long-term tissue compatibility and the potential need for removal even years after implantation [35,36]. In contrast, the more pronounced degradation of polylactide caps, transitioning from transparent to opaque and becoming brittle, suggests a faster integration with the surrounding tissue, which might favor natural healing processes but provides a shorter duration of mechanical protection. These findings align with previous studies with poly(DL-lactide-ε-caprolactone) nerve guides, which found no macroscopical residue after a two year follow up. However, histology revealed residual fragments, which could lead to secondary foreign body reactions [37].

This underscores the need for long-term studies to confirm the observed degradation patterns and for further research evaluating tissue responses, ideally including quantitative assessments of cap stability, integration and foreign body reactions. Immunohistochemical staining with markers such as CD68 for macrophage infiltration could help characterise the inflammatory response elicited by the cap materials and determine whether the observed degradation patterns are associated with a sustained foreign body reaction.

### Neuroma shape and adhesion

The survey conducted among surgeons revealed moderate to poor interrater reliability in classifying neuroma shapes and adhesion grades. This variability underscores the subjective nature of visual assessments in clinical practice. Nevertheless, the consistency of neuroma shape findings between our macroscopic evaluations and the surgeon survey suggests that both chitosan and polylactide caps tend to produce bulbous neuromas, which are generally easier to manage surgically and less painful in patients compared to the atypical neuromas with peripheral extensions seen in the Sham and Coagulation groups [31].

The adhesion data tentatively supports the benefit of caps, although the poor interrater reliability and the very limited sample size of Masson’s trichrome-stained histological sections greatly limit the robustness of these findings. Surgical observations during explantation suggest that the caps reduce direct connective tissue attachment to nerves, potentially simplifying surgical interventions for neuroma removal. However, the survey results indicate that chitosan-based caps appear to induce more perineural adhesions compared to the other groups. Longitudinal histological sections suggest that this adhesion consists predominantly of dense collagen surrounding the nerve tissue rather than intraneural scarring, which was observed in the other groups. The Sham group, in particular, exhibited intermingling of collagen and nerve tissue toward the distal end. Notably, although the section from the Polylactide group demonstrated a clearly delineated neuroma end, intermingling collagen fibers were also present more proximally.

If corroborated by further studies, this absence of direct contact between surrounding tissue and the nerve, as observed most clearly in the Chitosan Cap group, may aid in preventing painful neuroma formation. This notion is supported by extensive literature suggesting a correlation between tissue infiltration and contracting scar tissue around a neuroma, and the development of problematic symptoms [6,7,38,39]. Future studies incorporating immunohistochemical markers such as α-SMA for contractile myofibroblasts, which are associated with contracting scar tissue [40,41], could help quantify the extent of problematic intraneural intermingling across groups and determine whether the caps function as a genuine physical barrier against fibroblast infiltration, thereby strengthening the rationale for cap-based strategies in neuroma prevention.

### Histomorphometry

Histomorphometric analysis confirmed that the cap-treated groups had higher axon density and thicker myelin sheaths compared to the Sham group, indicating enhanced and more organized nerve regeneration. These findings are consistent with the literature on nerve regeneration, where biomaterials have been shown to increase myelinization and promote more effective nerve repair [21–23]. The significant differences in axon diameter and g-ratio between the Chitosan and Polylactide groups relative to the Sham group further highlight the potential of these materials to improve the quality of regenerated nerves.

Mechanistically, chitosan and its degradation products have been shown to enhance nerve regeneration by promoting Schwann cell proliferation [42] and by facilitating the formation of Bands of Büngner, which serve as structural guidance channels for organized axonal regrowth [23,43,44]. These processes are likely contributors to the improved histomorphometric outcomes observed in the present study. However, as immunohistochemical analyses such as S100 staining for Schwann cell presence [42] were not performed, the improved myelination in cap-treated groups reflects the downstream outcome of regenerative processes rather than direct evidence of enhanced Schwann cell activity. While these findings, taken together with the existing literature, strongly suggest that the above mechanisms underlie the observed results, confirmation requires dedicated immunohistochemical verification in future studies.

Notably, the Coagulation group showed no significant differences from the cap groups in axon density and myelin sheath thickness, suggesting that coagulation may also be effective in supporting nerve regeneration. One possible explanation is that thermal coagulation destroys and shrinks the perineural tissue, creating a sealed environment at the nerve stump that, similar to a physical cap, limits connective tissue invasion and preserves the local regenerative milieu (Zohar et al. 1996; Rummings et al.) However, whether the underlying mechanisms are truly equivalent remains to be established.

Our study adds to the growing body of evidence supporting the use of biomaterial-based interventions in nerve repair. Previous studies have demonstrated the benefits of chitosan in promoting nerve regeneration through its biocompatibility and ability to support Schwann cell activity [21–23]. Similarly, polylactide has been recognized for its biodegradability and capacity to guide axonal growth [26,45,46]. However, our findings provide a direct comparison between these materials and traditional nerve coagulation, offering a clearer understanding of their relative advantages and limitations. The trend toward comparable efficacy observed for nerve coagulation with nerve caps in certain parameters highlights the need for a nuanced approach in clinical decision-making.

In comparison to studies focusing on surgical removal of neuromas, our results suggest that nerve caps might offer a more sustainable solution by preventing the initial formation of disorganized nerve growth. The challenge remains in balancing the mechanical protection provided by the caps with their potential metabolic demands and long-term biocompatibility. Coagulation, being a simpler and less resource-intensive method, may still be preferable in settings where these factors are a concern, yet further studies are needed to confirm the trends seen in our findings.

### Limitations

A key limitation of this study is the relatively small sample size, which restricted the statistical approaches and prevented robust multiple comparison corrections. Post-hoc analyses, however, provide additional context regarding the strength of the observed effects. Large effect sizes were observed for t-testing between axon density and myelin sheath thickness in comparisons between the Sham and both Cap groups (Cohen’s d range: 1.27–1.82; observed power 0.66–0.92), supporting the robustness of these findings despite limited sample sizes. Similarly, ANOVA-based effect sizes (Cohen’s f: axon density = 0.56, axon diameter = 0.51, myelin sheath thickness = 0.71, and g-ratio = 0.42) indicate large overall group effects, with observed power ranging from 47–92%. In contrast, comparisons with small effect sizes and low power, such as for t-testing between Cap groups and the Coagulation group, should be interpreted cautiously. These analyses emphasize that while the present study provides important preliminary insights into neuroma morphology and nerve cap effects, further studies with larger cohorts are necessary to confirm these trends and allow for more robust multiple comparison testing.

Assessing pain in animal studies is inherently limited, as pain is subjective and animals cannot directly report it. This study focused on histological and macroscopic outcomes, which, when interpreted alongside current literature, allowed for potential correlations with pain. However, methods such as the Tinel test or tactile hyperalgesia by pinprick have been proposed to assess neuropathic pain in rats [47], and incorporating such approaches in future work will provide additional insight into neuroma-associated pain.

A further limitation is the lack of comparison to newer and more complex strategies such as TMR and RPNI, which represent important developments in the field. A direct comparison with these techniques would have exceeded the scope of this study but may provide promising avenues for future research.

### Clinical implications and future directions

While nerve caps represent a significant advancement in nerve repair technology, their practical application in clinical settings requires careful consideration of patient-specific factors, including the extent of nerve damage, possible metabolic impacts, and long-term outcomes. The findings from this study advocate for personalized treatment approaches that weigh the benefits and limitations of each method. Coagulation, due to its simplicity and trends towards comparable efficacy, may remain a viable option in many clinical scenarios, pending confirmation by more robust and larger-scale studies.

Future research should focus on long-term clinical studies with direct pain assessments to evaluate the sustained efficacy and biocompatibility of chitosan and polylactide nerve caps in humans. Additionally, exploring the metabolic responses and potential systemic effects of these biomaterials will be crucial in optimizing their use for nerve repair. Investigations into optimizing the coagulation technique to further enhance its outcomes could also be beneficial.

In conclusion, our study demonstrates that both chitosan and polylactide nerve caps are effective in promoting organized nerve regeneration and preventing disorganized neuroma formation, with distinct advantages over traditional nerve coagulation methods. However, the seen trend of comparable efficacy of nerve coagulation suggests that it remains a valuable technique, especially in contexts where simplicity and lower systemic impact are prioritized. These findings contribute valuable insights into the optimization of surgical treatments for peripheral nerve injuries, supporting the development of more effective and sustainable therapeutic strategies.

## Supporting information

S1 Table Quantitative data points. These tables contain the quantitative data points underlying the results presented in the main manuscript.
(PDF)

S1 Fig Masson’s Trichrome stained longitudinal sections. Full longitudinal sections stained with Masson’s Trichrome for each experimental group, from which the higher-magnification insets displayed in Fig 3 were derived. Sham (A), Coagulation (B), Chitosan Cap (C), Polylactide Cap (D).
(TIF)

S2 Fig Osmium tetroxide stained transverse sections of the Sham group. Full transverse sections stained with osmium tetroxide from the Sham group (a–h), used for quantitative analysis of axon density, axon diameter, myelin sheath thickness, and g-ratio.
(TIF)

S3 Fig Osmium tetroxide stained transverse sections of the Coagulation group. Full transverse sections stained with osmium tetroxide from the Coagulation group (a–h), used for quantitative analysis of axon density, axon diameter, myelin sheath thickness, and g-ratio.
(TIF)

S4 Fig Osmium tetroxide stained transverse sections of the Chitosan Cap group. Full transverse sections stained with osmium tetroxide from the Chitosan Cap group (a–h), used for quantitative analysis of axon density, axon diameter, myelin sheath thickness, and g-ratio.
(TIF)

S5 Fig Osmium tetroxide stained transverse sections of the Polylactide Cap group. Full transverse sections stained with osmium tetroxide from the Polylactide Cap group (a–h), used for quantitative analysis of axon density, axon diameter, myelin sheath thickness, and g-ratio.
(TIF)
