# Supplementary material for: Comparative efficacy of nerve coagulation vs. cap implantation in neuroma prevention using the rat sciatic nerve model: A histological and neuroradiological insight
Source: PLoS One. 2026 Aug 14;21(8):e0355057. doi: 10.1371/journal.pone.0355057 (PMC13475867; doi:10.1371/journal.pone.0355057)
Supplement: Supplementary file 1 [file pone.0355057.s001.pdf]

Group and animal number	Sham 1	Sham 2	Sham 3	Sham 4	Sham 5	Sham 6	Sham 7	Sham 8	Sham 9	Sham 10
Randomized number	34	7	5	23	22	6	9	35	26	20
**Histomorphometric results**										
Axon density [axons/mm²]	2535.78	3736.43	16126.25	4375.09	16327.84	8549.81	4919.19	7694.55	8205.6	
Axon diameter [µm]	1.4198	1.5924	1.4445	1.6185	1.4537	1.4813	1.4145	1.5523	1.6485	
Myelin sheath thickness [µm]	0.7415	0.6811	0.6561	0.6715	0.6044	0.7405	0.7492	0.8018	0.6886	
G-Ratio	0.4979	0.5329	0.5356	0.539	0.5346	0.4861	0.4763	0.4806	0.5262	
**Survery results: neuroma formation (1 - bulbous, 2 - fusiform, 3 - atypical)**										
NF-Rater 1	3	2	3	3	3	1	3	2	3	3
NF-Rater 2	2	3	3	3	3	1	3	2	3	3
NF-Rater 3	2	3	3	3	3	3	3	2	3	3
NF-Rater 4	3	3	3	3	3	3	3	3	3	3
NF-Rater 5	3	3	3	3	3	3	3	3	3	3
NF-Rater 6		1	1	2	2	1	2		2	2
NF-Rater 7	3	2	3	3	3	3	3	2	3	3
NF-Rater 8	2	3	3	3	2	2	3	2	2	3
NF-Rater 9	2	3	3	3	3	1	3	2	3	3
NF-Rater 10	3	3	3	3	3	1	3	3	3	3
NF-Rater 11	2	3	3	3	3	3	3	3	3	3
NF-Rater 12	1	2	2	3	3	1	2	1	3	2
NF-Rater 13	3	3	3	3	3	3	3	3	3	3
NF-Rater 14	2	2	3	3	3	1	2	1	3	2
NF-Rater 15	3	2	3	3	3	1	3	3	3	3
**Survery results: adhesions (0 - absent or thin, 1 – mild, 2 – moderate and 3 – severe)**										
A-Rater 1	2	3	2	2	2	1	1	2	1	3
A-Rater 2	1	3	2	1	2	1	2	2	1	3
A-Rater 3	2	2	2	2	2	1	2	2	1	2
A-Rater 4	1	3	1	1	2	1	1	1	1	3
A-Rater 5	2	3	2	3	3	1	2	2	1	3
A-Rater 6	2	3	2	2	2	1	2	1	1	3
A-Rater 7	1	3	2	1	2	0	1	1	1	2
A-Rater 8	0	3	3	1	3	1	3	1	0	3
A-Rater 9	1	2	3	2	3	2	2	2	2	3
A-Rater 10	1	2	1	1	0	1	0	1	0	0
A-Rater 11	2	2	2	2	2	0	2	2	1	2
A-Rater 12	1	1	0	1	2	0	2	1	1	1
A-Rater 13	1	3	1	1	0	0	0	2	1	1
A-Rater 14	0	2	1	2	0	0	2	1	1	1
A-Rater 15	2	3	3	2	3	2	2	2	2	3
A-Rater 16	1	2	1	2	1	1	2	1	2	2

Group and animal number	Coagulation 1	Coagulation 2	Coagulation 3	Coagulation 4	Coagulation 5	Coagulation 6	Coagulation 7	Coagulation 8	Coagulation 9	Coagulation 10
Randomized number	2	4	14	24	38	3	29	19	25	32
**Histomorphometric results**										
Axon density [axons/mm²]	4819.18	8523.22	11869.38	13382.43	11676.46	9561.84	28579.04	22849.64		14948.44
Axon diameter [µm]	1.7379	1.5232	1.4502	1.3631	1.7645	1.2646	1.6275	1.4286		1.6751
Myelin sheath thickness [µm]	0.7069	0.8733	0.7304	0.6763	0.8356	0.6606	0.707	0.7074		0.8051
G-Ratio	0.5563	0.4552	0.5079	0.4883	0.5061	0.4774	0.52	0.4853		0.4936
**Survery results: neuroma formation (1 - bulbous, 2 - fusiform, 3 - atypical)**										
NF-Rater 1	3	3	3	1	3	3	3	3	2	3
NF-Rater 2	3	3	3	2	3	2	3	3	2	3
NF-Rater 3	3	3	3	2	3	3	3	3	3	3
NF-Rater 4	3	3	3	3	3	3	3	3	3	3
NF-Rater 5	3	3	3	3	3	3	3	3	3	3
NF-Rater 6	3	3	2	3		2		2	2	
NF-Rater 7	3	3	3	1	3	3	3	3	3	3
NF-Rater 8	3	3	3	2	3	3	3	3	3	3
NF-Rater 9	2	3	3	2	3	3	3	3	3	3
NF-Rater 10	2	3	3	3	3	3	3	3	3	3
NF-Rater 11	3	3	3	3	3	3	3	3	3	3
NF-Rater 12	3	2	2	3	2	3	3	3	3	3
NF-Rater 13	3	3	3	3	3	3	3	3	3	3
NF-Rater 14	2	3	3	1	3	3	2	3	3	3
NF-Rater 15	3	3	3	3	3	3	3	3	3	3
**Survery results: adhesions (0 - absent or thin, 1 – mild, 2 – moderate and 3 – severe)**										
A-Rater 1	2	2	2	2	1	1	2	2		2
A-Rater 2	2	3	3	3	3	1	2	2		2
A-Rater 3	2	2	1	3	2	1	2	2		2
A-Rater 4	2	2	3	2	1	1	1	1		1
A-Rater 5	2	3	2	3	2	2	2	3		2
A-Rater 6	3	3	3	2	2	2	2	1		2
A-Rater 7	1	3	3	1	2	1	2	2		2
A-Rater 8	3	2	2	2	2	2	2	1		1
A-Rater 9	2	3	3	3	2	2	2	2		2
A-Rater 10	2	2	2	0	2	1	0	2		0
A-Rater 11	1	3	3	2	1	2	1	3		2
A-Rater 12	1	1	2	2	1	1	2	1		1
A-Rater 13	0	3	2	1	1	0	1	2		1
A-Rater 14	1	3	2	1	0	1	1	1		0
A-Rater 15	2	3	2	3	1	3	2	3		2
A-Rater 16	1	2	2	2	1	1	2	3		2

Group and animal number	Chitosan 2	Chitosan 3	Chitosan 4	Chitosan 5	Chitosan 6	Chitosan 7	Chitosan 8	Chitosan 9	Chitosan 10
Randomized number	16	21	28	31	17	37	39	13	8
**Histomorphometric results**									
Axon density [axons/mm²]	12356.9	23279.09	5638.89		16599.0417	10482.73	26630.33	20315.48	11508.84
Axon diameter [µm]	1.8852	1.7342	1.7913		1.3959	1.759	1.7232	1.4183	1.8155
Myelin sheath thickness [µm]	0.769	0.7964	0.822		0.7785	0.8407	0.72	0.8405	0.897
G-Ratio	0.5367	0.5189	0.5184		0.4643	0.4967	0.5491	0.4486	0.4901
**Survery results: neuroma formation (1 - bulbous, 2 - fusiform, 3 - atypical)**									
NF-Rater 1	1	2	2	1	1	1	1	1	2
NF-Rater 2	1	2	1	1	1	1	1	1	2
NF-Rater 3	3	2	1	1	1	1	1	1	2
NF-Rater 4	1	2	2	1	1	1	1	1	2
NF-Rater 5	3	2	2	1	1	1	1	2	2
NF-Rater 6	2	3			1			3	3
NF-Rater 7	1	2	1	1	1	1	1	2	2
NF-Rater 8	2	2	1	1	1	1	2	2	1
NF-Rater 9	2	2	1	1	1	1	1	1	2
NF-Rater 10	1	2	2	1	1	1	1	1	2
NF-Rater 11	2	3	2	1	1	1	1	1	2
NF-Rater 12	1	1	1	1	1	1	1	1	2
NF-Rater 13	1	2	2	1	1	1	1	1	2
NF-Rater 14	2	1	1	1	1	1	1	1	1
NF-Rater 15	3	1	1	1	1	1	1	1	2
**Survery results: adhesions (0 - absent or thin, 1 – mild, 2 – moderate and 3 – severe)**									
A-Rater 1	1	3	3	3	3	1	2	3	2
A-Rater 2	1	2	2	3	3	2	2	1	3
A-Rater 3	2	3	3	3	3	3	3	3	3
A-Rater 4	2	3	1	3	3	2	3	1	3
A-Rater 5	2	3	2	3	3	2	2	3	3
A-Rater 6	2	3	2	3	3	1	3	2	2
A-Rater 7	3	3	3	3	3	2	3	2	2
A-Rater 8	2	2	3	3	3	1	2	2	3
A-Rater 9	3	3	2	2	3	2	2	3	2
A-Rater 10	0	0	1	2	0	0	2	0	2
A-Rater 11	2	3	1	3	3	3	2	3	3
A-Rater 12	2	2	2	3	3	2	2	2	2
A-Rater 13	1	1	0	2	1	1	0	0	0
A-Rater 14	2	3	1	3	3	1	2	2	3
A-Rater 15	2	3	2	3	3	1	2	3	3
A-Rater 16	2	2	2	2	2	1	2	2	2

Group and animal number	Polylactide 1	Polylactide 2	Polylactide 3	Polylactide 4	Polylactide 5	Polylactide 6	Polylactide 7	Polylactide 8	Polylactide 9	Polylactide 10
Randomized number	10	33	11	36	27	12	30	18	1	15
**Histomorphometric results**										
Axon density [axons/mm²]	21221.6582	14726.096	22353.1	16336.58		12752.5	13558.49	11267.77	17480.28	11218.56
Axon diameter [µm]	1.5291	1.486	1.5244	1.575		1.5776	1.6066	1.6933	1.3389	1.5795
Myelin sheath thickness [µm]	0.7292	0.7544	0.8453	0.776		0.7554	0.7945	0.8685	0.7592	0.7899
G-Ratio	0.4977	0.4994	0.465	0.4919		0.4955	0.485	0.4773	0.4597	0.4796
**Survery results: neuroma formation (1 - bulbous, 2 - fusiform, 3 - atypical)**										
NF-Rater 1	1	2	2	1	1	1	1	1	1	1
NF-Rater 2	1	3	2	1	1	1	1	1	1	1
NF-Rater 3	1	2	2	1	1	1	1	1	1	1
NF-Rater 4	2	2	2	1	3	1	1	1	1	1
NF-Rater 5	2	2	2	2	1	1	1	1	2	1
NF-Rater 6	1		1		1	2		2	1	1
NF-Rater 7	1	2	1	1	1	1	1	1	1	1
NF-Rater 8	2	2	1	1	1	1	1	1	2	1
NF-Rater 9	1	2	1	1	1	1	1	1	1	1
NF-Rater 10	2	2	2	1	1	1	1	1	1	1
NF-Rater 11	2	3	2	1	2	1	1	1	2	1
NF-Rater 12	1	1	1	1	1	1	1	1	1	1
NF-Rater 13	2	3	2	1	1	1	1	1	2	1
NF-Rater 14	2	2	1	1	1	1	1	1	1	2
NF-Rater 15	1	2	2	1	1	1	1	1	1	1
**Survery results: adhesions (0 - absent or thin, 1 – mild, 2 – moderate and 3 – severe)**										
A-Rater 1	2	3	3	0	2	2	3	2	2	1
A-Rater 2	2	3	1	1	3	0	2	2	1	2
A-Rater 3	3	3	3	2	3	1	2	2	1	2
A-Rater 4	2	2	3	1	3	2	2	3	1	2
A-Rater 5	2	3	2	1	3	1	2	2	1	2
A-Rater 6	1	3	3	1	3	1	3	1	2	2
A-Rater 7	2	3	3	2	3	2	3	2	2	2
A-Rater 8	2	3	3	1	3	0	2	3	1	1
A-Rater 9	2	3	3	0	1	1	1	2	1	2
A-Rater 10	0	2	0	0	2	0	0	1	1	0
A-Rater 11	3	3	3	2	3	2	3	2	2	2
A-Rater 12	2	3	3	1	3	1	2	1	0	1
A-Rater 13	2	1	1	2	2	1	2	2	0	2
A-Rater 14	3	1	2	1	1	0	2	2	2	1
A-Rater 15	3	3	3	1	3	2	3	2	1	1
A-Rater 16	2	2	2	1	2	1	2	2	1	2

MRI Data													
Diameter [mm]					Length [mm]					Circularity			
Sham	Coagulation	Chitosan	Polylactide		Sham	Coagulation	Chitosan	Polylactide		Sham	Coagulation	Chitosan	Polylactide
1.282	1.442	1.415	1.329		7.323	4.798	0.961	0.784		0.262	0.359	0.772	0.715
1.534	1.359	1.489	1.027		5.266	3.134	1.292	1.055		0.399	0.57	0.808	0.714
1.574	1.326	2.127	1.78		4.055	3.572	1.536	1.835		0.413	0.551	0.773	0.714
2.138	1.166	1.774	1.857		6.469	2.624	1.014	1.348		0.316	0.519	0.721	0.791
1.051	1.733	2.428	2.02		3.451	2.572	0.758	1.504		0.453	0.584	0.562	0.776
1.412	0.996	2.061	1.715		5.851	2.832	1.145	1.147		0.349	0.467	0.723	0.768
	1.073	1.938	2.017			2.047	1.218	0.875			0.572	0.754	0.69
	1.213	1.369	1.791			2.646	1.056	1.381			0.52	0.749	0.802
	1.391	1.934	1.867			3.251	1.238	0.953			0.521	0.744	0.698
	1.36					2.769					0.555		
